# Arthroscopic Subacromial Decompression in Patients With Subacromial Impingement Syndrome

**DOI:** 10.7759/cureus.70569

**Published:** 2024-09-30

**Authors:** Rahul Salunkhe, Vishal S Patil, Mohammed Talha Muneer, Sahil Chowdhary, Shirsha Ray

**Affiliations:** 1 Orthopaedics, Dr. D. Y. Patil Medical College, Hospital and Research Centre, Dr. D. Y. Patil Vidyapeeth (Deemed to be University), Pune, IND; 2 Orthopaedics and Trauma, Joint Replacement Surgery, Dr. D. Y. Patil Medical College, Hospital and Research Centre, Pune, IND; 3 Orthopaedic Surgery, Dr. D. Y. Patil Medical College, Hospital and Research Centre, Dr. D. Y. Patil Vidyapeeth (Deemed to be University), Pune, IND

**Keywords:** arthroscopic decompression, ases score, constant score, rotator cuff, shoulder function, subacromial impingement syndrome

## Abstract

Background: Subacromial impingement syndrome (SIS) is a prevalent cause of shoulder dysfunction, affecting a significant portion of the adult population. It is associated with considerable pain, functional limitations, and disability. The evolution of treatment options, including arthroscopic subacromial decompression (ASAD), necessitates an updated evaluation of clinical outcomes and functional improvements.

Objective: This study aims to assess the effectiveness of ASAD in patients with SIS by evaluating clinical signs and functional outcomes at three, six, and 12 months postsurgery.

Methods: A prospective interventional study was conducted from August 2022 to November 2023 at Dr. D. Y. Patil Medical College, Hospital & Research Centre, Pune, India. Twenty-five patients diagnosed with SIS were included. Baseline sociodemographic and clinical characteristics were recorded. Patients underwent arthroscopic decompression, and functional outcomes were measured using the Constant score and American Shoulder and Elbow Surgeons (ASES) score at three, six, and 12 months. Data were analyzed using Statistical Product and Service Solutions (SPSS, version 21.0; IBM SPSS Statistics for Windows, Armonk, NY), with a significance level set at p<0.05.

Results: The study cohort comprised 64% females and 36% males, with a mean age of 46-55 years. The majority (72%) were engaged in labor work. The mean body mass index (BMI) was 24.89. Rotator cuff status was intact in 56% of patients, while 44% had partial tears. At baseline, 84% reported pain upon lifting the arm, and 92% experienced loss of motion. Postsurgery, the Constant-Murley score improved significantly from 36% poor at baseline to 92% excellent at 12 months. Similarly, the ASES score increased from a baseline mean of 17-84.9 by 12 months (p<0.01). The acromiohumeral distance increased from 9.7 mm before treatment to 10.4 mm after treatment (p=0.009). No infections or neurological deficits were reported.

Conclusion: ASAD significantly improves shoulder function and reduces symptoms in patients with SIS. The Constant-Murley and ASES scores demonstrate substantial improvement over a 12-month follow-up period. The procedure is associated with favorable outcomes and minimal complications, supporting its effectiveness as a treatment modality for SIS.

## Introduction

Shoulder dysfunction primarily leads to sub-acromial impingement syndrome (SIS), which is the main reason for health problems among adults [1]. It is associated with around two-thirds of all shoulder pathologies, resulting in considerable pain, loss of function, and disability [2]. SIS is characterized as a progressive, degenerative rotator cuff disease, where the soft tissues become entrapped [2]. Initially, the etiology of SIS was thought to be primarily due to extrinsic factors. This results in impingement, which causes the narrowing of the space beneath the acromion. However as the result has progressed, apoptosis a component within the body, has been planned to play a major role in the growth of rotator cuff tendinopathy. The condition may be advanced by alterations in the rotator cuff tendons inside the body [2,3]. Patients who are affected usually have chronic pain in the absence of any previous traumatic events. The affected end causes discomfort and pain while elevating their arms and making forced overhead movements [4].

The diagnosis of SIS is primarily clinical, meaning that it is predicated on the physical examination results, as well as the patient's complaints [3]. Imaging methods are further diagnostic tools that are utilized for verification of diagnosis and to find out the reason for discomfort. For example, plain radiographs can be very informative; a narrowed acromiohumeral distance seen on a radiograph often indicates rotator cuff tendinopathy or tear [2,3].

SIS is primarily diagnosed clinically, with imaging modalities to find differential diagnoses and additional reasons for shoulder discomfort [3]. A reduced acromiohumeral distance suggests rotator cuff tendinopathy or a tear; therefore, plain radiographs are useful in this context. With the help of CT arthrography and MRI scans, the rotator cuff tendon might be effectively evaluated [3].

At present, SIS has no universally definitive management, also approaches to therapy frequently vary depending on doctors' and patients' choices. From non-invasive techniques to invasive procedures and multitude of healing methods were documented in medical journals. Surgical possibilities encompass mini-open procedures, potentially involving cuff repair or acromioplasty, depending on the individual case [3,5].

In the past, an anterior acromioplasty procedure was recommended, which involved the removal of the coracoclavicular ligament through an open surgical approach [2]. As technology and surgical tools have progressed, doctors address SIS by popular methods such as acromioplasty by arthroscopic method, alternatively termed ASAD, had given positive results and is deemed equivalent to open approaches for managing SIS [5-7].

Given these advancements, we suggest carrying out a prospective analysis to assess the clinical signs in SIS patients and the functional clinical outcomes after arthroscopic subacromial decompression.

## Materials and methods

Before the start of the study, clearance was obtained from the Institute Ethics Committee. This study was a descriptive, prospective study conducted at the Orthopedics Department of DY Patil Medical College and Hospital. It included all patients attending the department during the study period who were diagnosed with subacromial impingement and met the inclusion and exclusion criteria. The study took place from August 2022 to November 2023, with data collection spanning 15 months and data analysis and reporting conducted over six months. The sample size, calculated using an online tool for comparing two independent means, required 25 participants, based on a population standard deviation of 2.2, a 95% confidence interval, and a precision of 1. Consecutive patients meeting the criteria were informed about the study, and those who agreed were recruited following institutional ethics approval.

Data were collected using a predesigned and pretested proforma, which included clinical examination details, operative notes, and discharge certificates. Patients underwent a comprehensive baseline sociodemographic and clinical evaluation, including hematological and radiological assessments (X-rays, ultrasonography, and MRI). For patients with bilateral symptoms, the most affected shoulder was selected for primary intervention. Outcome parameters were recorded at follow-ups at three, six, and 12 months using the same proforma.

The inclusion criteria were patients aged 18-55 years with shoulder symptoms lasting between six months and three years, normal passive glenohumeral movement, rotator cuff tear, frozen shoulder, or impingement syndrome. Patients with impaired glenohumeral joint rotation, a history of acute trauma, previous surgery or fracture near the affected shoulder, or known osteoarthritis in the acromioclavicular or glenohumeral joints were excluded.

The intervention for the subacromial decompression group involved an arthroscopic examination of the glenohumeral joint, rotator cuff, and subacromial bursa under general anesthesia, followed by bursectomy and partial resection of the antero-inferior part of the acromion and the coracoacromial ligament. Patients were advised to perform light arm movements within pain limits before discharge. Stitches were removed by general practitioners after 10 days, and physiotherapists guided patients in active exercises, including rotator cuff muscle strengthening.

Outcomes were evaluated at three, six, and 12 months post-intervention using the Constant-Murley score, which includes sub-scores for pain (measured on a visual analogue scale), limitations in daily activities, active range of motion in four shoulder directions, and force/strength, as well as the ASES (American Shoulder and Elbow Surgeons) score, a 100-point scale assessing pain and performance in daily activities. Data were entered into Microsoft Excel (Microsoft® Corp., Redmond, WA), checked for accuracy, and analyzed using Statistical Product and Service Solutions (SPSS, version 21.0; IBM SPSS Statistics for Windows, Armonk, NY). Continuous variables were expressed as mean ± standard deviation (SD), while categorical variables were reported as frequencies (percentages). Descriptive summaries and chi-square tests were used to assess differences between proportions, and continuous variables were compared using Student's t-test. A p-value of <0.05 was considered statistically significant.

## Results

The study comprised 25 subjects, with a gender distribution of 36% male and 64% female (Table 1).

**Table 1:** Gender distribution among the study subjects

Gender	N	%
Male	9	36
Female	16	64
Total	25	100

The majority of participants were in the 46-55 age group, accounting for 48% of the subjects, followed by 36% in the 36-45 age group and 16% in the 18-35 age group (Table 2).

**Table 2:** Age distribution among the study subjects

Age Group (in years)	N	%
18-35	4	16
36-45	9	36
46-55	12	48
Total	25	100

Regarding the duration of complaints, 48% of the subjects reported symptoms lasting more than 12 months, 36% had symptoms for 6-12 months, and 16% had symptoms for less than six months (Table 3).

**Table 3:** Duration of complaints among the study subjects

Duration	N	%
<6 months	4	16
6-12 months	9	36
>12 months	12	48
Total	25	100

The occupation distribution revealed that 72% of the subjects were engaged in labor work, while 28% were involved in non-labor work (Table 4).

**Table 4:** Occupation among the study subjects

Occupation	N	%
Labour work	18	72
Non-labour work	7	28
Total	25	100

The mean body mass index (BMI) of the subjects was 24.89 with a standard deviation (SD) of 3.21 (Table 5).

**Table 5:** BMI among the study subjects

Variables	Value
Mean	24.89
SD	3.21

Regarding the condition of the rotator cuff, 56% of the subjects had an intact rotator cuff, while 44% had a partial tear (Table 6).

**Table 6:** Rotator cuff among the study subjects

Rotator cuff	N	%
Intact	14	56
Partial tear	11	44
Total	25	100

The most common complaints among the subjects were loss of motion, reported by 92%, and pain upon lifting the arm, reported by 84% (Table 7).

**Table 7:** Complaints among the study subjects

Variables	N	%
Complaints of Pain Upon Lifting the Arm	21	84
Loss of Motion	23	92

The American Shoulder and Elbow Surgeons (ASES) score showed significant improvement over time, with a baseline mean score of 17.02 (SD: 4.60) that increased to 49.46 (SD: 8.13) at three months, 68.71 (SD: 9.27) at 6 months, and 84.95 (SD: 7.80) at 12 months (Table 8). All intervals showed statistically significant improvements with a p-value of <0.01.

**Table 8:** ASES score among the study subjects at different intervals ASES: American Shoulder and Elbow Surgeons *Statistically significant improvements

ASES Score	Mean	SD
Baseline	17.02	4.60
3^rd^ Month	49.46	8.13
6^th^ Month	68.71	9.27
12^th^ Month	84.95	7.80
p value		
Baseline vs 3^rd^ Month	<0.01*	
Baseline vs 6^th^ Month	<0.01*	
Baseline vs 12^th^ Month	<0.01*	

The acromiohumeral distance also showed a statistically significant increase after treatment, with the mean distance rising from 9.71 (SD: 1.23) before treatment to 10.47 (SD: 0.96) after treatment (Table 9).

**Table 9:** Acromiohumeral distance before and after the treatment *Statistically significant improvements

Interval	Mean	SD	p value
Before	9.71	1.23	0.009*
After	10.47	0.96	

The Constant-Murley score, which evaluates shoulder function, showed considerable improvement over time. At baseline, 64% of the subjects were rated as "poor" and 36% as "fair," but by the 12th month, 92% were rated as "excellent" and 8% as "good" (Table 10).

**Table 10:** Constant score among the study subjects

Interval	Excellent		Good		Fair		Poor	
	N	%	N	%	N	%	N	%
Baseline	0	0	0	0	16	64	9	36
3^rd^ Month	7	28	13	52	4	16	1	4
6^th^ Month	16	64	8	32	1	4	0	0
12^th^ Month	23	92	2	8	0	0	0	0
p value	<0.01*							

Notably, no complications such as infections or neurological deficits were reported during the study.

## Discussion

Our prospective interventional study analyzed the sociodemographic characteristics and clinical presentations of patients with subacromial impingement syndrome. We also compared the changes in baseline parameters at three, six, and 12 months following arthroscopic decompression, using Constant-Murley and ASES scores.

Demography

In our study, the majority of patients were aged 46-55 years (48%), followed by 36-45 years (36%), and 72% were engaged in labor work. In comparison, David et al. analyzed the value of physiotherapy in subacromial impingement syndrome (SIS) patients, involving 16,813 patients with an average age of 47 years [8]. Siron et al. evaluated the results of arthroscopic subacromial decompression (ASAD) in managing SIS in a prospective study of 28 patients with an average age of 55.14 years [9]. Mikkel et al. studied conservative management in 129 SIS patients, 98 of whom underwent conservative treatment with a six-month follow-up (average age: 56 years) [10]. The age distribution in our study is consistent with these studies, although our follow-up period extended to one year after the arthroscopic procedure. Ravi Kiran et al. examined ASAD in 20 SIS patients with an average age of 38 years, ranging from 20 to 65 years, whereas our study included patients aged 18-55 years [11]. Rahul et al. analyzed ASAD's efficacy in a cohort of 73 patients with a mean age of 56 years [12]. Bidwai et al. compared ASAD and small open repair for medium-sized rotator cuff injuries in 42 patients, with an average age of 64 years in the ASAD group [7]. Beard et al. conducted a study on subacromial shoulder pain and ASAD treatment involving 313 patients, with average ages of 52.9, 53.7, and 53.2 years in different treatment groups [13]. Haahr et al. compared arthroscopic decompression and exercises in SIS patients aged 18-55 years [14]. Van Holsbeeck et al. evaluated outcomes of open decompression (OD) and arthroscopic decompression (AD) in 53 patients in each group, with mean ages of 49.9 years (OD) and 38.7 years (AD) [15].

In our study, 64% of the 25 patients were female. This contrasts with David et al., where males comprised 51% of the study population [8]. In Siron et al., 39% of the subjects were male, while Mikkel et al. reported 52% female participation [9,10]. Ravi et al. observed 60% male participants, while Rahul et al. noted a female predominance [11,12]. In Bidwai et al., males were the majority (11:3) [7]. Beard et al. reported that females constituted 51%, 50%, and 50% of the decompression, arthroscopy-only, and no-treatment groups, respectively [13]. Van Holsbeeck et al. found that males comprised 58.5% of the OD group and 53.2% of the AD group [15].

Duration of symptoms

In our study, 48% of patients had symptoms lasting more than 12 months. Additionally, 84% reported pain upon lifting the arm, and 92% experienced a loss of motion. Ravi et al. reported an average symptom duration of 4.95 months, while Rahul et al. found that patients experienced shoulder pain for at least six months [4,12]. Haahr et al. observed that patients had rotator cuff disorders with symptoms lasting between six months and three years [14]. Van Holsbeeck et al. reported mean symptom durations of 27.1 months (AD) and 25.5 months (OD) [15]. Our study's results align with these findings, with symptom durations consistently exceeding one year.

Scoring system assessment

Christiansen et al. reported that 55% of women with lower education levels utilized physiotherapy less [8]. Within the first hospital visit, 43% received physiotherapy, and 21% received more extensive treatment. Postsurgery, 80% received physiotherapy, with 52% undergoing more extensive treatment. Physiotherapy exercises were performed by 65% after the first visit and 84% post-operation. In Mikkel et al., 75% of patients received physiotherapy during follow-up, 87% performed home exercises, and they spent an average of 1,040 minutes on these exercises [10]. Ravi et al. found that primary SIS patients received six weeks of physiotherapy without improvement [11]. Our study, however, did not document the effect of physiotherapy on postoperative patients.

In our study, the mean BMI was 25.8. Additionally, 56% of patients had intact rotator cuffs, while 44% had partial tears. The acromiohumeral distance significantly improved from 9.7 mm before treatment to 10.4 mm after treatment. Siron et al. reported a 57% intact rotator cuff rate and a 43% partial tear rate, with acromiohumeral distance increasing from 9.77 mm preoperatively to 10.28 mm postoperatively [9].

Constant score

At baseline, 36% of patients had a poor Constant-Murley score, which improved to 52% at three months. By six months, 64% of patients had an excellent Constant-Murley score, and by the end of one year, 92% had an excellent score. No infections or neurological deficits were observed. Nizam Siron et al. found that 80% of patients achieved good or excellent Constant-Murley scores six months postsurgery [9]. Rahul et al. reported median Constant-Murley scores of 39 preoperatively and 67 at six months, with significant improvement [12]. Bidwai et al. observed worse Constant-Murley scores in patients with re-rupture on ultrasound compared to those with intact repairs [7]. Haahr et al. noted an average Constant-Murley score increase from 34.8 (physiotherapy group) and 33.7 (decompression group) at baseline to 57.0 and 52.7, respectively, after 12 months [14]. Our study's results align with these findings, showing consistent improvement in Constant-Murley scores over the follow-up period.

ASES score

In our study, the mean ASES score improved significantly following arthroscopy, from 17 at baseline to 49.4 at 3 months, 68.7 at six months, and 84.9 at 12 months. Siron et al. reported an ASES score increase from 16.07 before surgery to 88.21 six months post-surgery [9]. Mikkel et al. observed a similar trend, with pain variables decreasing and Shoulder Pain and Disability Index (SPADI) scores improving over time [10]. Rahul et al. reported that 76.5% of patients returned to work, and 79.4% resumed leisure activities, although our study did not document postoperative recovery times [12]. Bidwai et al. noted improvements in Constant-Murley scores, ASES, and DASH scores in the cuff repair group compared to the ASAD group, though not statistically significant [7]. David et al. found minimal advantages in surgical groups over no treatment, likely due to different study comparisons [9]. Holsbeeck et al. reported good to excellent outcomes in 81.1% of the OD group and 83.1% of the AD group [15].

Limitations

This study focused on patients aged 18-55 years, limiting the generalizability of findings to older populations. Therefore, the effect of the intervention on the elderly remains unknown.

## Conclusions

We assessed the impact of arthroscopic decompression among the subacromial impingement syndrome patients. We documented around 56% with intact rotator cuff. In conclusion, we determined that the constant score improved from the baseline to good in around half of the patients at three months. By the end of the follow-up, 92% had an excellent constant score. None of the patients had an infection or neurological deficit following the treatment. Post-procedure, the baseline mean ASES score increased from 17 to 84.9 by the end of the one-year follow-up. We documented a statistically significant increase in ASES scores following the arthroscopic decompression.

This study demonstrates that ASAD is an effective treatment for SIS, offering substantial improvements in shoulder function and a significant reduction in symptoms over a 12-month follow-up period. The progressive enhancement in Constant-Murley and ASES scores underscores the procedure’s efficacy, with the majority of patients achieving excellent outcomes by the end of the study. Additionally, the procedure was associated with minimal complications and no reports of infections or neurological deficits. These findings support ASAD as a valuable surgical option for managing SIS, particularly in patients with persistent symptoms and functional limitations.

## References

[1] Vecchio P, Kavanagh R, Hazleman BL, King RH (1995). Shoulder pain in a community-based rheumatology clinic. Br J Rheumatol.

[2] Dong W, Goost H, Lin XB (2015). Treatments for shoulder impingement syndrome: a PRISMA systematic review and network meta-analysis. Medicine (Baltimore).

[3] Dorrestijn O, Stevens M, Winters JC, van der Meer K, Diercks RL (2009). Conservative or surgical treatment for subacromial impingement syndrome? A systematic review. J Shoulder Elbow Surg.

[4] Garving C, Jakob S, Bauer I, Nadjar R, Brunner UH (2017). Impingement syndrome of the shoulder. Dtsch Arztebl Int.

[5] Milano G, Grasso A, Salvatore M, Zarelli D, Deriu L, Fabbriciani C (2007). Arthroscopic rotator cuff repair with and without subacromial decompression: a prospective randomized study. Arthroscopy.

[6] Biberthaler P, Beirer M, Kirchhoff S, Braunstein V, Wiedemann E, Kirchhoff C (2013). Significant benefit for older patients after arthroscopic subacromial decompression: a long-term follow-up study. Int Orthop.

[7] Bidwai AS, Birch A, Temperley D, Odak S, Walton MJ, Haines JF, Trail I (2016). Medium- to long-term results of a randomized controlled trial to assess the efficacy of arthoscopic-subacromial decompression versus mini-open repair for the treatment of medium-sized rotator cuff tears. Shoulder Elbow.

[8] Christiansen DH, Frost P, Frich LH, Falla D, Svendsen SW (2016). The use of physiotherapy among patients with subacromial impingement syndrome: impact of sex, socio-demographic and clinical factors. PLoS One.

[9] Nizam Siron K, Mat Lani MT, Low CL, Kow RY (2021). Arthroscopic subacromial decompression in the treatment of shoulder impingement syndrome: a prospective study in Malaysia. Cureus.

[10] Clausen MB, Merrild MB, Witten A, Christensen KB, Zebis MK, Hölmich P, Thorborg K (2018). Conservative treatment for patients with subacromial impingement: changes in clinical core outcomes and their relation to specific rehabilitation parameters. PeerJ.

[11] Ravi Kiran HG, Siddique PA, Adarsh T, Vijay C, Mruthyunjaya Mruthyunjaya, Supreeth N (2017). Functional outcome of arthroscopic subacromial decompression in primary shoulder impingement syndrome due to extrinsic mechanical causes. Int J Orthop Sci.

[12] Bhattacharyya R, Edwards K, Wallace AW (2014). Does arthroscopic sub-acromial decompression really work for sub-acromial impingement syndrome: a cohort study. BMC Musculoskelet Disord.

[13] Beard DJ, Rees JL, Cook JA (2018). Arthroscopic subacromial decompression for subacromial shoulder pain (CSAW): a multicentre, pragmatic, parallel group, placebo-controlled, three-group, randomised surgical trial. Lancet.

[14] Haahr JP, Østergaard S, Dalsgaard J (2005). Exercises versus arthroscopic decompression in patients with subacromial impingement: a randomised, controlled study in 90 cases with a one year follow up. Ann Rheum Dis.

[15] Van Holsbeeck E, DeRycke J, Declercq G, Martens M, Verstreken J, Fabry G (1992). Subacromial impingement: open versus arthroscopic decompression. Arthroscopy.

